# End-expiratory esophageal pressure versus lower inflection point in acute lung injury

**DOI:** 10.1186/cc12041

**Published:** 2013-03-19

**Authors:** A Yaroshetskiy, D Protsenko, E Larin, O Ignatenko, B Gelfand

**Affiliations:** 1 Russian National Research Medical University, Moscow, Russia; 2 City Hospital #7, Moscow, Russia

## Introduction

No recommendations available concerning protocols of static PV loop and esophageal pressure measurements use set positive end-expiratory pressure (PEEP). The aim of the study was evaluation of the significance of the lower inflection point (LIP) and esophageal pressure monitoring for PEEP adjustment in ALI and ARDS.

## Methods

A prospective study performed in one general ICU. We include 72 patients who received mechanical ventilation before evaluation and met ARDS criteria by AECC (1994) - acute onset, PaO_2_/FiO_2 _lower than 250 Torr, bilateral infiltrates on chest X-ray. Exclusion criteria were age <15 years and pregnancy. We drew a static pressure-volume loop with sustained inflation 40×30 (PV loop) for all patients using a lowflow technique (Hamilton G5) and measured the esophageal pressure (Avea) in 36 of 72 patients. After that PEEP was set according to zero end-expiratory transpulmonary pressure. We compare PV loop data with esophageal pressure measurements.

## Results

The low inflection point median was 8 (95% CI = 5 to 10.5) mbar, which does not correspond to the empirically set optimal PEEP of 13 (95% CI = 12 to 15) mbar (*P <*0.001, Wilcoxon test). End-expiratory esophageal pressure (EEEP) median was 14 (95% CI = 12 to 18) mbar, the correlation between LIP and EEEP was poor (ρ = 0.279, *P *= 0.049). We find significant correlation between static compliance and EEEP (ρ = -0.421, *P *= 0.005). Sustained inflation did not lead to improved oxygenation (*P >*0.05). PEEP adjustment by EEEP led to an increase in PaO_2_/FiO_2 _- median 107 mmHg (95% CI = 18 to 147, *P <*0.001). EEEP was similar to empirically set PEEP (*P >*0.05).

## Conclusion

LIP has poor correlation with EEEP. PEEP adjustment by esophageal pressure was close to empirically set PEEP and can improve oxygenation.

